# Heart transplantation at 50+: Celebrations and challenges

**DOI:** 10.21542/gcsp.2018.10

**Published:** 2018-06-30

**Authors:** Magdi Yacoub, Asghar Khaghani

**Affiliations:** 1 Imperial College London, London, UK; 2 Michigan State University, College of Human Medicine, Grand Rapids, Michigan, USA

This year marks the 51st anniversary of the first human heart transplant operation by Christiaan Barnard at the Groote Schuur Hospital in Cape Town. This event has had a profound effect on Science, Medicine and Humanity, almost beyond expectations. The anniversary calls for celebrations, and evaluation, as with maturity comes accountability. We here attempt to summarise the reasons for celebrations, and highlight the remaining many challenges and expectations.

Regarding the celebrations, this anniversary exemplifies and calls for celebration of at least four human attributes; courage, innovation, compassion, and human spirit.

## Courage

According to Sir Winston Churchill, courage is the most important human attribute, if present, all other desirable attributes follow. The definition of Courage, given by Sir Winston, is “to do what you think is right, even if you know it could harm you”. This applies to many acts in life, and performing the first heart transplant is certainly an example. Progress in science is achieved through a series of imaginative leaps, to be followed by scrutiny. Thus, the initial enthusiasm for heart transplantation was followed by extensive debate and criticism (refutations) resulting in a ‘moratorium’ for a period of time.

## Innovation

Creativity or innovation is essential for progress, and has been defined by Sir Peter Medawar as “producing ostensibly, out of nothing, something of beauty, order or Significance”. With all the benefits of Science, Medicine, and Humanity, heart transplantation is a strong reminder of the need for innovation. It also shows the importance of being conscious of finding an answer to the question “innovation at what price?” to patients and Society.

Following a period of intensive debate, studies aimed at establishing the risk benefit ratio and cost effectiveness of heart transplantation has helped to establish and refine the procedure. Formulating rules and regulations for innovative medicine, while essential, should not be restrictive. A constant open dialogue between scientists and Society should be strongly encouraged, to resolve such issues as brain death criteria in adults, infants with anencephaly, and presumed consent.

## Compassion

Another important human attribute to celebrate is compassion. This is manifest in society, welcoming the concept of organ donation, as a gift of life. However, the application of these principles are extremely variable and needs to be addressed. This applies to the global application of modern medicine, and specifically cardiac surgery.

### Taking cardiac surgery to the people

Extensive discussions of this topic took place at the Celebrations of the 50th anniversary of the first heart transplantation, organised by Dr Peter Zilla and his colleagues in Cape Town. This meeting was attended by more than 200 participants, representing professional societies, industry, and academic and popular press. A detailed plan for addressing the unacceptable inequalities was formulated, and the “Cape Town Declaration” will be published, and hopefully implemented, in the very near future.

## Human spirit

Another closely related reason for celebrating is “human spirit”, which is both unifying and combines many of the attributes discussed above. This was clearly obvious during the Cape Town meeting.

## Challenges

The future of cardiac transplantation depends on solving the current challenges, none of them insuperable. The four main challenges include scarcity of donor organs, chronic rejection, complications of immunosuppressive drugs, and gross inequalities in global access to transplantation.

### 1. Scarcity of donor organs

Currently, this constitutes the most important limitation to organ transplantation, with a wide variation in the numbers for organ donation, varying from 0-35 per million in different countries. In addition, a very high number of donated organs (up to 70 percent) are simply not used, due to inadequate care of potential donors. Efforts to deal with these problems are being made through increasing public awareness, introduction of new legislations, such as presumed consent, and adoption of tested programmes like the ‘Spanish model’. The use of donors after circulatory death (DCD) is also being explored and is gradually expanding. The use of methods to enhance donor preservation, including *ex vivo* perfusion of donor organs, is very promising and needs to be explored further

#### Xenotransplantation

In theory, xenotransplantation offers the opportunity of having an unlimited number of donor organs, and therefore continues to to be the focus of intensive research. The use of primates is ethically unacceptable, and carries the risks of introducing a very large number of simian viruses. In contrast, the use of hearts from genetically modified pigs, was thought to be ethically justifiable up until recently. However the risk of transmitting, the integrated pig retrovirus PERV, which carries a small but definite risk of being transmitted to and from the recipient, remains a major problem. The recent introduction of gene editing with CRISPR-Cas9 technology has had a dramatic effect in this field. The same technology may now be used to prevent rejection of the xenografts.

#### Tissue engineering

Understanding the molecular two way communication between the extracellular matrix and different types of cells, coupled with the development of different types of intelligent scaffolds designed to instruct the appropriate type of cell, is being used for engineering cardiac tissue. This approach could produce myocardial patches, to replace small parts and possibly the entire heart. The experimental use of entire decellularised allogenic or xenogenic hearts, capable of beating (albeit for limited periods of time) following reperfusion with blood or different cocktails of cells, provides unique opportunities for research.

### 2. Chronic rejection

This continues to be responsible for a major part of the the constant risk hazard of mortality and morbidity following cardiac transplantation. Understanding of the mechanisms involved, coupled with advances in immunosuppressive therapy, has tended to reduce the incidence of this dreaded complication.

The use of balloon dilatation, stents or bypass grafts for established cases has given disappointing results due to the diffuse nature of the disease.

Retransplantation is rarely used, carries a significantly higher risk than the initial operation, and could be argued to be unethical due to the scarcity of donor organs.

Induction of Medawar’s specific immune tolerance is gradually being developed and carries the hope of a definitive solution to this problem.

### 3. Complications of immunosuppressive drugs

The increased incidence of severe infections, cancer, and kidney failure, constitute another strong reason for intensifying research efforts to develop the long-awaited Medewar’s specific immune tolerance.

## Conclusions

The last 5o years has witnessed a considerable expansion in the use of heart transplantation resulting in benefit to Humanity and Science. The next 50 years hold the promise of further expansion and benefit, provided the challenges already identified, and partially solved, can be effectively dealt with.

